# Long-Read Sequencing of Human Cytomegalovirus Transcriptome Reveals RNA Isoforms Carrying Distinct Coding Potentials

**DOI:** 10.1038/s41598-017-16262-z

**Published:** 2017-11-22

**Authors:** Zsolt Balázs, Dóra Tombácz, Attila Szűcs, Zsolt Csabai, Klára Megyeri, Alexey N. Petrov, Michael Snyder, Zsolt Boldogkői

**Affiliations:** 1 Department of Medical Biology, Faculty of Medicine, University of Szeged, Szeged, 6720 Hungary; 2 Department of Genetics, School of Medicine, Stanford University, Stanford, California, 94305 USA; 3 Department of Medical Microbiology and Immunobiology, Faculty of Medicine, University of Szeged, Szeged, 6720 Hungary; 4 Department of Structural Biology, School of Medicine, Stanford University, Stanford, California, 94305 USA; 5 Department of Biological Sciences, College of Sciences and Mathematics, Auburn University, Auburn, Alabama 36849 USA

## Abstract

The human cytomegalovirus (HCMV) is a ubiquitous, human pathogenic herpesvirus. The complete viral genome is transcriptionally active during infection; however, a large part of its transcriptome has yet to be annotated. In this work, we applied the amplified isoform sequencing technique from Pacific Biosciences to characterize the lytic transcriptome of HCMV strain Towne varS. We developed a pipeline for transcript annotation using long-read sequencing data. We identified 248 transcriptional start sites, 116 transcriptional termination sites and 80 splicing events. Using this information, we have annotated 291 previously undescribed or only partially annotated transcript isoforms, including eight novel antisense transcripts and their isoforms, as well as a novel transcript (RS2) in the short repeat region, partially antisense to RS1. Similarly to other organisms, we discovered a high transcriptional diversity in HCMV, with many transcripts only slightly differing from one another. Comparing our transcriptome profiling results to an earlier ribosome footprint analysis, we have concluded that the majority of the transcripts contain multiple translationally active ORFs, and also that most isoforms contain unique combinations of ORFs. Based on these results, we propose that one important function of this transcriptional diversity may be to provide a regulatory mechanism at the level of translation.

## Introduction

The Human Cytomegalovirus (HCMV) is a human pathogenic beta-herpesvirus that can cause life-threatening infections in new-born infants and immunocompromised patients. Congenital HCMV infections can lead to severe malformations or even death^1,2^. The genome of the HCMV is one of the largest in the *Herpesviridae* family, and its coding potential is not yet fully understood. Estimates of the number of its protein coding sequences had formerly ranged from 164^3–5^ to 220^6^, while a recent study identified 751 individual, translationally active open reading frames (ORFs) by ribosome profiling^7^. Most of these novel ORFs were short ORFs with potential regulatory functions, or N-terminally truncated versions of already annotated proteins. The HCMV genome contains a Unique Long (UL) region and a Unique Short (US) region, each bracketed by terminal and internal repeats (TRL/IRL, TRS/IRS, respectively)^8^. Laboratory HCMV strains such as the Towne strain, which was used in our experiments, have undergone substantial genetic alterations compared to the wild-type (wt) virus, which severely affected their pathogenicity^9^.

HCMV, similarly to other herpesviruses^10^, has a complex transcriptional architecture; alternative transcription initiation^11^, alternative splicing events^5,12^, and polycistronic transcripts^13^ all increase the coding potential of the viral genome. Alternative transcription initiation gives rise to the different isoforms of MIE^14^ and UL136 in HCMV^15^, it governs the differential expression of UL44 at early and late times of infection^11^, but the ubiquitous presence of alternative TSSs in the host^16^ and in other herpesviruses^17,18^ suggests that alternative TSSs may have an important role in the regulation of many more genes than what we are presently aware of. Even though splicing in herpesviruses is relatively rare^19^, over 100 splice junctions have been described in HCMV^5,7,12^ – many of which are alternatively spliced. Polycistronic mRNAs are not unique to prokaryotes; they are widespread in nematodes^20^ and have been detected in insects as well^21–23^. The expression of peptides from these transcripts can be regulated by UTR sequences or cellular factors^24,25^. Ribosome footprint analysis has identified hundreds of translationally active short ORFs in the HCMV genome^7^, which demonstrates that many transcripts express uORFs beside the main ORFs, and may implicate that even transcripts that were previously thought to be non-coding, could possibly have coding potential as polycistronic peptide coding RNAs (ppcRNAs). Although the importance of most of these short peptides continues to remain unknown, it has been suggested in HCMV and also in other organisms that short upstream ORFs may have a significant influence on gene-expression^26–28^.

Short-read sequencing analysis has demonstrated that the complete HCMV genome is transcriptionally active during lytic infection^5^. It has also revealed numerous splice sites, and confirmed many of the previously detected. The HCMV genome, however, continues to be insufficiently annotated^13^. Generating accurate transcript models using short-read sequencing data is challenging^29^, and methods with high specificity, such as Northern-blotting and rapid amplification of cDNA ends (RACE) techniques are too laborious to be used for mapping the transcriptome of complex organisms. On the other hand, long-read sequencing is capable of determining the base composition of full-length transcripts, which enables distinction between transcript isoforms and alternative splicing events, and thus renders it a powerful tool in transcript discovery^30^.

To date, among the major shortcomings of long-read sequencing methods are their low throughput and relatively high rate of error^31^. While the latter does not typically pose a challenge in transcriptomic analyses, the low coverage of larger genomes means that the analysis is at a greater disposition for picking up erroneous signals. RNA-degradation and template switching are both potential sources of errors. 5′-truncated RNAs are frequently seen as a result of RNA-degradation, which hinder the detection of alternative internal TSSs. Template switching can produce false transcript isoforms, such as false splice junctions or chimeric reads in a homology-dependent manner^32^.

In our study, we aimed to broaden our knowledge regarding the HCMV transcriptome through employing Pacific Biosciences (PacBio) Single Molecule Real-Time (SMRT) sequencing in order to characterize the HCMV RNA population in human fibroblast cells during lytic infection. Our focus was to identify novel transcripts, transcript isoforms, novel splice junctions, and to determine the coding potential of these transcripts, by comparing these to available ribosome profiling data.

## Results

### Data processing

**Figure 1:**
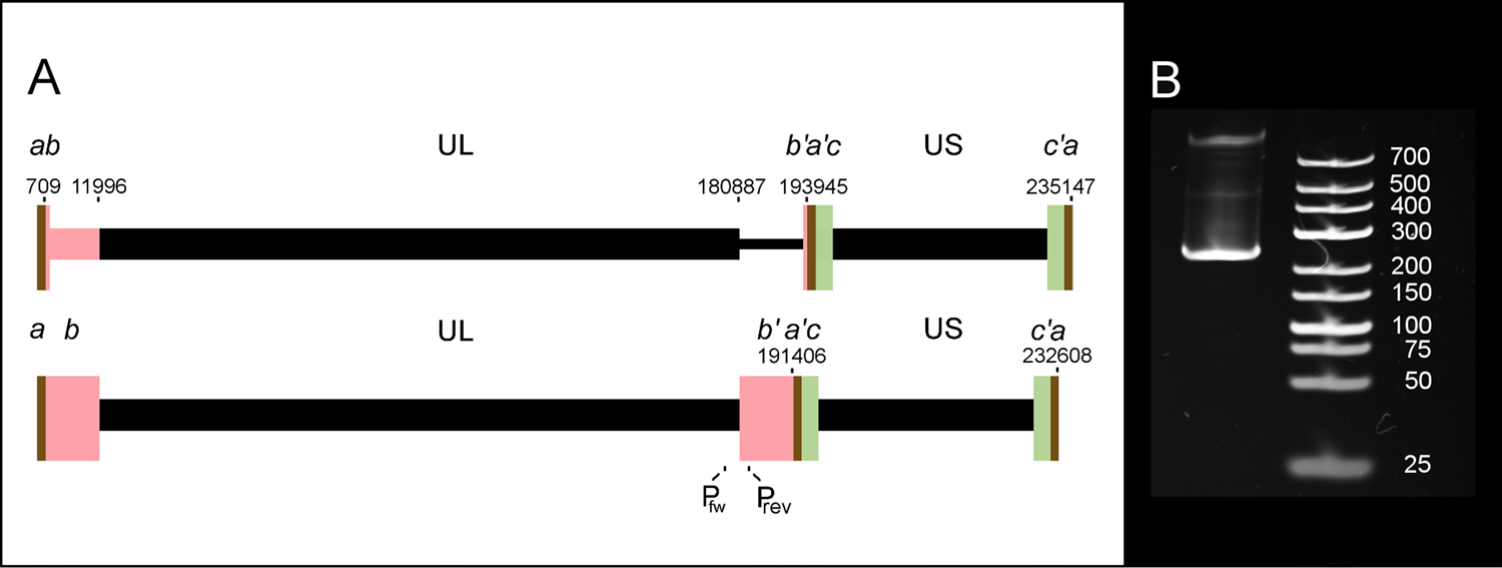
Genomic rearrangement of the HCMV isolate used in this study. Panel A shows schematic representations of the original Towne strain virus above) and the isolate used in our experiments (below). The unique long (UL) and unique short (US) sequences are bracketed by repeat sequences (**a**–**c**), marked by coloured rectangles (brown, pink and green, respectively). In our isolate, the UL*/b’* (180887–193945) region of the FJ616285 genome is substituted by the 710–11996 region. To confirm this rearrangement, primers have been designed to in the ends of the UL (P_fw_) and *b’* (P_rev_) regions 243 nt apart. Panel B shows the PCR product of approximately 250 nt. The original gel photo, from which Panel B was cropped is shown in Supplementary Fig. S1.

**Figure 2:**
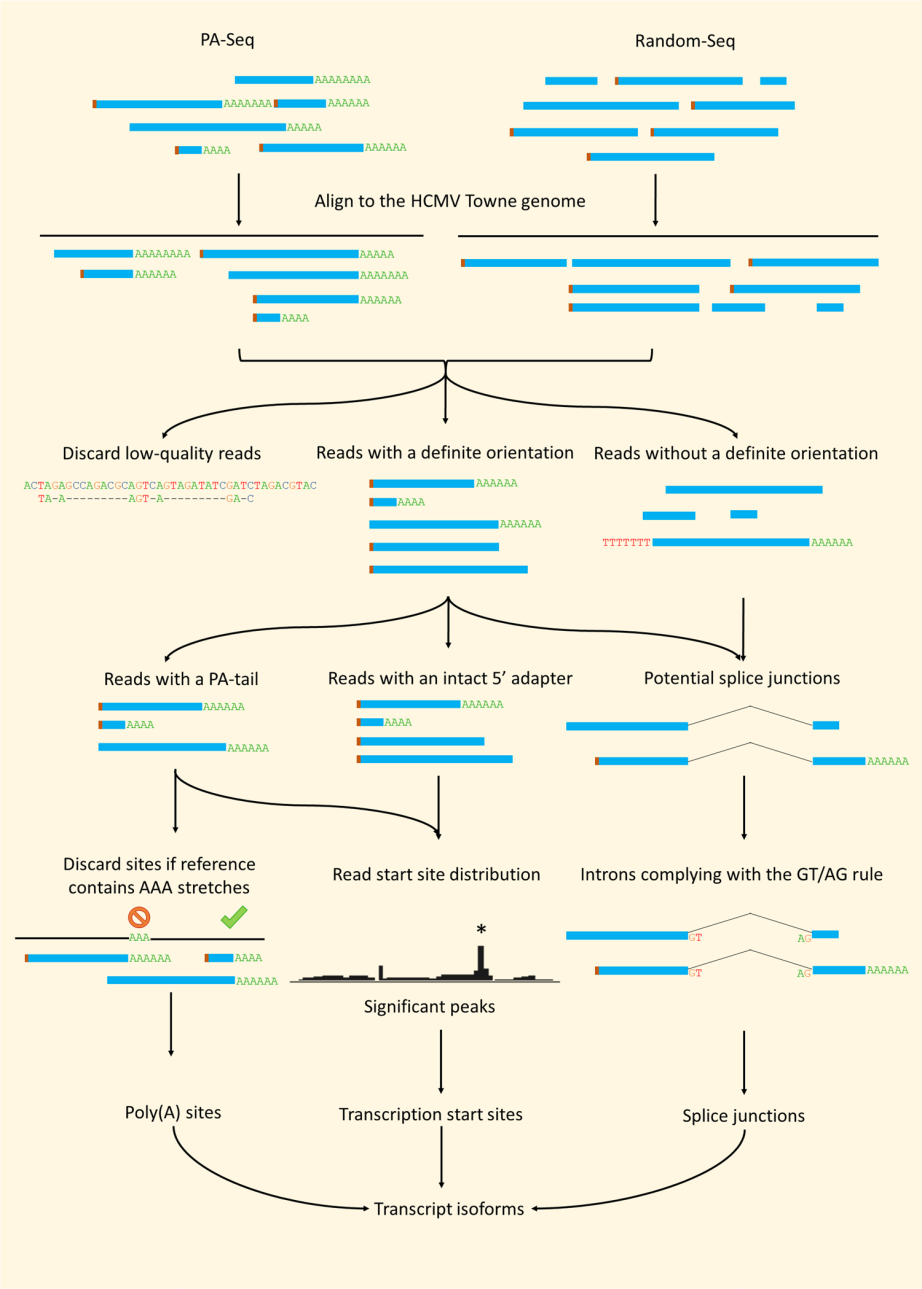
Transcriptome profiling pipeline. Long-consensus reads were aligned to the HCMV genome. Reads with a high mismatch or indel ratio (>5%) were discarded. All good quality reads were used to identify splice junctions. Only the deletions complying with the GT-AG rule were accepted as splice junctions. Reads containing 15 terminal (A) mismatches (i.e. a poly(A) tail) were considered for the validation of TESs. A TES was accepted as valid if two reads confirmed the same nucleotide position and the genomic region did not contain a stretch of 3 or more (A)s. Reads with a definite orientation were considered for the identification of TSSs. If the number of reads starting at a given genomic position was significantly higher than that would be expected according to the Poisson distribution, the genomic position was accepted as a TSS. Transcript isoforms were annotated based on reads containing the above mentioned annotated features.

HCMV total RNA was analysed in a mixed sample containing specimens from eight different post infection time points. Two libraries were prepared: one containing cDNAs of polyadenylated transcripts, reverse transcribed using oligo(dT) primers and the other containing cDNAs that were reverse transcribed using random primers. The HCMV Towne (FJ616285) genome was used to map the sequencing reads. Our reads showed a 13 kb long deletion at the UL133-UL145 region, combined with the duplication of the RL region (Fig. 1, Panel A), which is characteristic of the Towne varS^9^. The deletion was confirmed by PCR (Fig. 1, Panel B, and Supplementary Fig. 1). This deletion is similar to the one found in the AD169 strain^9^. The ATCC stock is reported to also contain an intact variant (varL), however, no sequencing reads mapped to the UL133-145 region, nor could this region be amplified by a specific primer sequence (not shown). Our pipeline for transcriptome profiling has been summarized in Fig. 2. From the poly(A)^+^ library 49,859 reads and from the random-primer-based library 3,233 reads aligned to the HCMV genome representing approximately a 255.8x average coverage of the genome. Reads with 5% or higher mismatch or indel error rates were excluded from the analysis. This meant 2,672 reads from the Poly(A)-selected and 156 reads from the random library. Read orientation could be determined based on the presence of a distinct poly(A) tail (i.e. the read contained at least 15 (A) mismatches at its 3′ end) or based on the presence of the 5′ adapter sequence. In the poly(A)^+^ library 94% (47,100 out of 49,859), while in the random-primer sequencing, 62% (1,995 out of 3,233) of the reads had a definite orientation. The reason for the lower percentage in the random library is that only 53 (1.6%) reads from that library contained a poly(A) tail, therefore the determination of read orientation was based almost exclusively on the presence of the 5′ adapter sequence.

### Transcription End Sites

**Figure 3:**
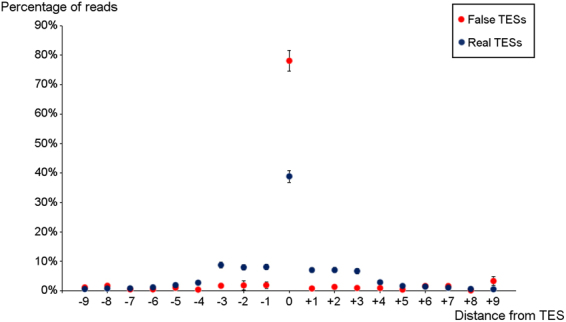
The spread of TESs. The scatter plot shows the frequency of reads ending in the vicinity of true TESs (blue) and that of false TESs (red), which are genomic locations containing stretches of 3 (A)s or more. Error bars represent standard errors.

The polyadenylated fraction of HCMV RNAs was used to determine the TESs. Oligo(dT) primers are considered specific for the detection of poly(A) tails, and therefore TESs; however, long stretches of (A)s, which can also bind the oligo(dT) primers (albeit with a lower affinity), and therefore produce nonspecific 3′-truncated transcripts. Beside the oligo(dT) primers binding to internal stretches of (A)s, template switching may also be capable of producing 3′-truncated transcripts in a homology-dependent manner. The sequence similarity between stretches of (A)s and the poly(A) tail make these genomic positions predilection sites for template switching. Indeed, we have found 290 potentially spurious TESs, where the genomic positions contained at least three (A)s (Supplementary Dataset S1). After discarding these positions, we were able to identify 116 TESs (Supplementary Dataset S2). It has been described in earlier studies^17,33^, and we were further able to observe that the exact positions of the polyadenylation sites of the transcripts varied by a few nucleotides. In our annotation, the nucleotide where the most reads terminated was designated as the TES. This spread showed a slight dependence from the type of the poly(A) signal (Supplementary Fig. S2), and could not be observed in the case of the false poly(A) sites, where reads ended uniformly at the same nucleotide. (Fig. 3). Unlike real TESs (70%), these positions were rarely (14%) preceded by the most common canonical poly(A) signal (AATAAA). We justify the cut-off values used by the our analysis by the fact that the discarded TESs were rarely preceded by the canonical poly(A) signal, while the accepted TESs were preceded by the canonical poly(A) signal as frequently as the host TESs were^33^.

### Transcription Start Sites

The applied PacBio isoform sequencing (IsoSeq) protocol allows for the precise identification of TSSs, however, 5′-degradation of the transcripts continues to be a critical issue. Assuming that sequencing reads start significantly more often at real TSSs than at other genomic positions, we looked for genomic positions where more reads started than it was to be expected in its 101-nt-long region (from 50 nt downstream to 50 nt upstream of the position). The Poisson probability distribution was used to assess significance, similarly to the approach used by Amman *et al*.^34^; the 101-nt window was chosen, in order to avoid the bias originating from the differential expression of genomic regions. 789 genomic positions were identified as local maxima (±10nt), where at least two reads started at the exact same nucleotide, 248 (Supplementary Dataset S3) of which were accepted as TSSs (p < 6.337*10^−5^, Bonferroni 0.05/789 = 6.337*10^−5^). Approximately half (121 out of 248) of the accepted TSSs are verified by reads in both libraries. The reason for this low verification rate is the low read count of the random library. Our analysis also confirmed many TSSs which were published in previous studies using 5′-RACE (as listed with references in Supplementary Dataset S4). Even though these studies often investigated HCMV strains other than Towne, the identified TSSs in this study and other studies often matched with base-pair precision.

### Novel splice junctions

80 splice junctions were identified when using the criteria of the presence of at least two independent reads and the occurrence of GT dinucleotide adjacent to the donor site, and AG adjacent to the acceptor site. 21 of these splice junctions have not been detected previously, but most of these novel splice junctions share a common donor or acceptor site with previously described splice junctions. Seven other frequent deletions in the reads that did not adhere to this GT-AG rule contained repetitive sequences of varying lengths (3–6 nucleotides) around the deleted segment, and therefore had probably arisen through the course of template switching (Supplementary Dataset S5). It is important to note that repetitive elements of similar lengths are found in 33 of the 80 accepted splice junctions as well (Supplementary Dataset S6), but 28 of these splice junctions have either been described previously or they share either donor or acceptor sites with previously described splice junctions. Forty-four (55%) of the detected splice junctions were confirmed by reads in both the poly(A) selected and in the random libraries. A similar portion (10 out of 21 [48%]) of the newly described splice junctions were detected in both libraries.

### Transcript annotation

**Figure 4:**
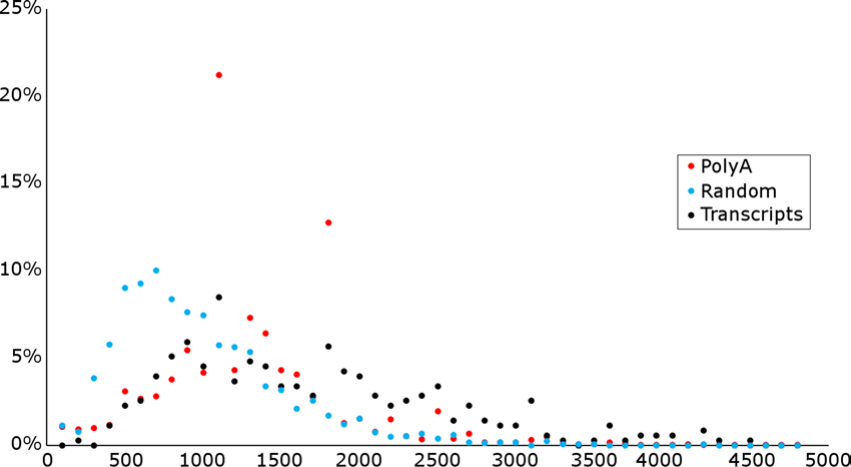
Read and transcript length distribution. The length distribution of reads aligning to the HCMV genome from the polyA selected (red) and random (blue) libraries are presented, together with the length distribution of the identified transcripts (black).

Altogether, 354 HCMV transcripts and transcript isoforms were identified (Supplementary Fig. S3); these include already known and novel transcripts, as well as previously unannotated polycistronic transcript variants, 5′-UTR and 3′-UTR isoforms and splice variants. The numbers of reads assigned to each transcript (read starts in the ±10-nt-bin around TSS of the transcript and ends at ±10-nt-bin around the TES of the transcript) is shown in Supplementary Dataset S4. Gatherer and colleagues^5^ have reported a very high expression rate of the RL4 gene, which was confirmed by our analysis; we obtained an approximately 45-fold average coverage in this genomic location (Supplementary Fig. S3). This has not been captured in the read counts assigned to the transcripts, because the RL4 region contains multiple false poly(A) sites, and reads ending in these false poly(A) sites were not assigned to any transcript. Most of the 291 newly described transcript variants are length variants of already known transcripts, containing different TSSs, TESs, splice junctions or a combination of these. In our analysis, we have also confirmed 63 previously annotated transcripts; the lack of confirmation for other transcript variants does not imply that they are not transcribed in the Towne strain. It could possibly mean that they are expressed at low levels or at other p.i. times (our samples contained an equal amount of RNA templates from the different p.i. time points, since viral transcription is more active at late times, it means that late viral transcripts are likely to be overrepresented). Sequencing read length also poses a limitation to transcript identification. The library preparation methods used in this study prefer cDNA sizes between 1 and 2 kbp (Fig. 4). This means that very short or very long transcripts could not be detected by this analysis.

### Transcript isoforms offer a means to translational regulation

**Figure 5:**
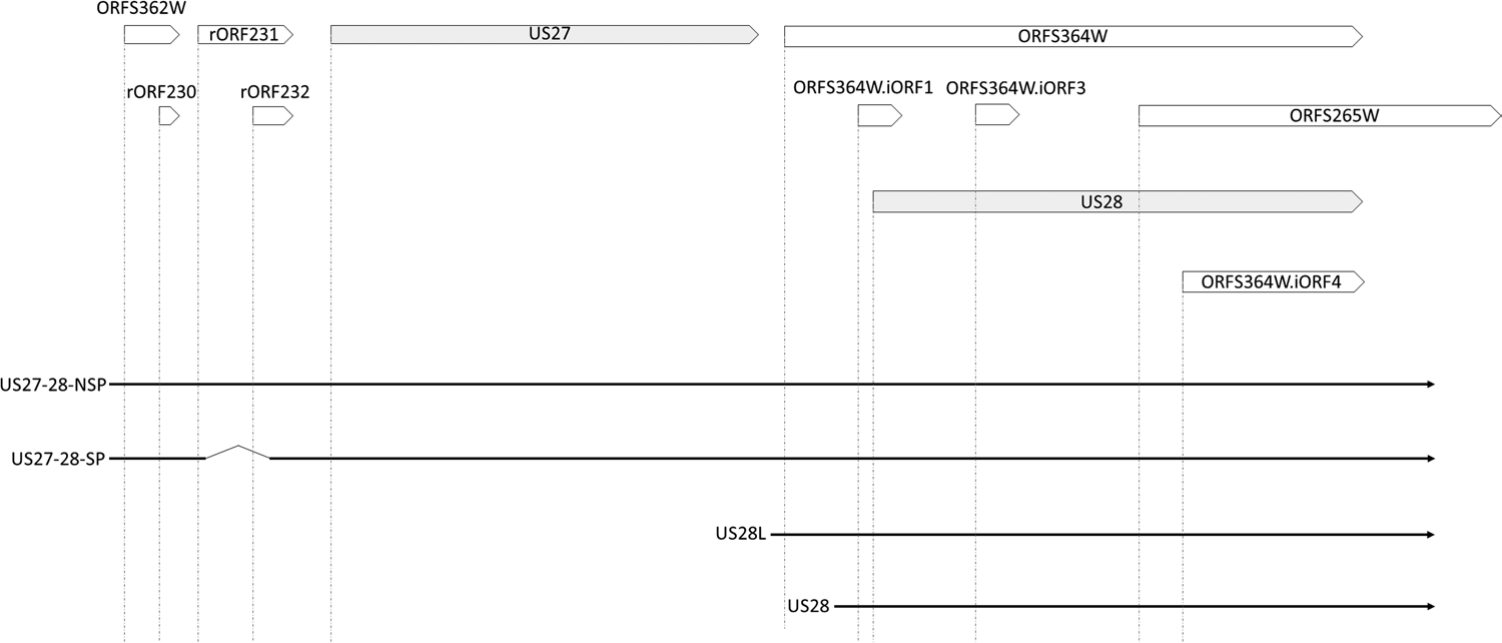
Transcript isoforms contain different ORFs. The figure shows an example of the differential peptide coding capacity (above) of transcript isoforms (below). Canonical ORFs are represented as arrows with a grey background, the other translationally active ORFs are represented as empty arrows and named as published by Stern-Ginossar *et al.* ^7^. Dotted vertical lines mark the translational start sites of the ORFs. The transcript isoforms of these two genes can be differentially translated due to polycistronism (US27-28 or only US28), alternative splicing (the splicing in US27 leads to the excision of 75 nucleotides and does not cause frameshift) or alternative transcription initiation (leading to a truncated protein in the cases of ORFS364W and US28).

Experimentally verified, translationally active ORFs^7^ were mapped to the identified transcripts to evaluate their coding potential (Supplementary Fig. S4). To allow for comparison between the two experiments, the identified transcripts were transferred to the strain Merlin genome based on sequence similarity. Of the 354 identified transcript isoforms, 314 could be transferred to the strain Merlin genome. 89.8% (282 out of 314) of the viral transcripts contained ORFs that were shown to be translationally active (Supplementary Dataset S7). Most of these transcript isoforms (206 of the 282 [73.1%]) were found to possess a unique coding potential, i.e. they contained unique ORF combinations. TSS-isoforms differed in the number of uORFs that preceded the main ORF, alternatively spliced isoforms and TSS-isoforms contained differently truncated versions of the same main ORF or contained multiple main ORFs (Fig. 5).

### Novel transcripts

**Figure 6:**
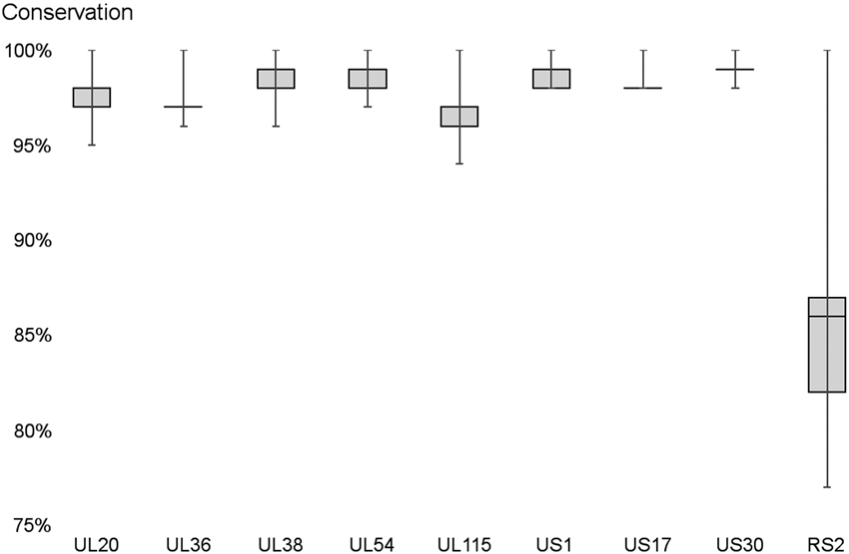
Conservation of the novel transcripts. The nucleotide sequences of the genomic regions corresponding to the longest transcript isoforms were aligned to publicly available HCMV genomes. The similarities of these regions in other genomes compared to the sequence in FJ616285 are depicted in a boxplot. The whiskers represent the range of the data. In some cases, the median and the quartile values are the same.

We have identified nine transcripts and their isoforms in the genomic regions where no transcripts have been described to date, and no canonical ORF had been annotated before (Supplementary Fig. S5). Eight of these transcripts are antisense to canonical ORFs (UL20, UL36, UL38, UL54, UL115, US1, US17 and US30). Kaye *et al*.^35^ have reported a transcriptional activity antisense to the UL16 mRNA, and have indicated a shorter transcript, partially overlapping with the UL20-AS1 therefore what they described, might have been a shorter variant of this transcript, but no overlapping transcript to the shorter variant, UL20AS2, has been described. The isoforms of UL20AS, UL54AS, US1AS and US17-AS all contain one or more short (<100AA), but translationally active ORFs. The isoforms of the UL36AS UL38AS UL115AS, and the US30AS, transcripts contain only short ORFs and these ORFs have not shown significant ribosome coverage in previous experiments^7^. Our analysis also discovered a novel transcript in the short repeat region (designated RS2), which is partially antisense to the RS1. Although ribosome profiling experiments detected no transcriptionally active ORFs in this transcript, it does contain two short ORFs, one well conserved, overlapping with the RS1 ORF, and one less conserved, that is not antisense to the RS1 ORF (Supplementary Fig. S5). The nucleotide conservation of the novel transcripts in other HCMV genomes is presented in Fig. 6. The transcripts which are antisense to known coding genes, are highly conserved, however, the transcript RS2, which is only partially antisense to the RS1 gene, is significantly less conserved (p < 10^−5^, analysis of variance).

## Discussion

The data presented herein demonstrates the utility of long-read sequencing for transcriptome profiling of small-genome organisms, such as viruses. We have isolated and reverse transcribed total RNAs from HCMV infected lung fibroblast cells at various phases of the replication cycle of the virus. We used two library preparation methods for PacBio sequencing in order to survey the transcriptional activity along the HCMV genome. We were able to detect a deletion event combined with the duplication in the viral genome similar to the deletion in strain AD169^36,37^, as already described in strain Towne varS^9^. However, we did not detect the intact varL variant, which is also reported to be present in the ATCC HCMV strain Towne virus stock.

We have developed a pipeline for transcriptome profiling of long-read RNA sequencing data. We have described 291 novel transcript isoforms, and an additional 13 novel TSSs, 22 novel TESs and 11 novel splicing events. We have described important caveats of cDNA sequencing and proposed methods to exclude potential erroneous signals that may arise during reverse-transcription. We do not rule out the possibility that some of the excluded TESs or splice sites truly function as such, but because of the high probability of template switching occurring at these loci, they should be regarded with suspicion and can only be proven by methods that eliminate the possibility of template switching. We believe that it is important to note that as few as three consecutive genomic (A)s already predispose to false TES identification. This is an important caveat and to our best knowledge, this is the first report about this issue, while previously only the possibility of the oligo(dT) primer annealing to long (approximately 20 nt) stretches of (A)s has been considered as a potential source of false TESs^38^. False splice site detection (another potential error originating from template switching) can be controlled by the GT-AG rule (Chambon’s rule)^39^ to some extent. However, it is possible that some of the putative splice junctions that adhere to the GT-AT rule have arisen through template switching.

We found that the majority of the described isoforms contain a unique combination of ORFs. Often, the difference between the length isoforms is that the shorter isoform encodes an N-terminal truncated version of the same protein, which may then have an altered effector function^11^. In other cases, the longer isoforms incorporate an extra uORF upstream of the main ORF, and as such might regulate the expression of that protein^27^. It is also possible, nevertheless, that the measured transcript diversity is only transcriptional noise stemming from cryptic promoters or an error-prone transcription apparatus. Further investigations are needed to interpret the biological significance of these findings.

We have also described nine novel transcripts and their isoforms, in genomic regions of HCMV where no transcript has been previously described. As all of these transcripts are at least partially antisense to already known transcripts, their functions may simply be regulatory, but since they also express shorter ORFs, which are found in other HCMV strains as well, they may have an important function as ppcRNAs. Since the UL38AS transcript contains a long (1043 nt), sequence without stop codons in one frame, it is possible that it might code for a long protein molecule initiated by a non-canonical start codon. Such an UL38A protein might explain the diverse functions of the UL38 locus^40,41^. All the antisense transcripts are highly conserved among the HCMV strains. The low conservation of the RS2 transcript, especially the part that is not antisense overlapping with RS1, might indicate that its ORFs are not functional.

In summary, our data substantially increases the number of HCMV transcripts and combine our annotation with previous data in order to propose a possible function of transcriptional diversity in the virus. Knowing the full transcriptional repertoire of the virus is essential for studying its gene expression; long-read RNA sequencing allows for the precise characterization of transcript isoforms. Joining such data with the information gained through the application of other methods could potentially go a long way in improving our knowledge of the regulation of gene expression.

## Materials and Methods

### Cells and viral infection

HCMV strain Towne (ATCC VR-977) was grown at 37 °C and 5% CO_2_ in human lung fibroblast cells [MRC-5; American Type Culture Collection (ATCC)] in DMEM supplemented with 10% FBS (Gibco Invitrogen), and 100 μl penicillin-streptomycin 10 K/10 K mixture (Lonza) per ml. Rapidly-growing semi-confluent MRC-5 cells were infected with a multiplicity of infection (MOI) of 0.05 plaque-forming units (pfu)/cell, followed by incubation under the same conditions as above. The infected cells were incubated for 1 h, the virus suspension was then removed and washed with PBS. Fresh culture medium was then added to the cells, which were incubated for 1, 3, 6, 12, 24, 72, 96 or 120 h. All experiments were performed in accordance with relevant guidelines and regulations.

### cDNA library synthesis and SMRTbell template preparation

A total RNA mixture containing samples in equal volume from all post infection (p.i.) time points was isolated using the NucleoSpin^®^ RNA kit (Macherey-Nagel). The Oligotex mRNA Mini Kit (Qiagen) was used to select polyadenylated RNAs and RiboMinus™. The Eukaryote System v2 (Ambion) kit was used on the RNA samples for random primer-based sequencing. Adapter-linked anchored oligo(dT) primers and random primers were used for the reverse transcription (RT) of the Poly(A)-selected and the rRNA-depleted samples, respectively. The cDNA was prepared following the PacBio Iso-Seq protocol, using the Clontech SMARTer PCR cDNA Synthesis Kit. The cDNA was amplified through 18 cycles. 500ng of amplified cDNA sample was used to prepare the SMRTbell libraries using the PacBio DNA Template Prep Kit 2.0.

### Single Molecule Real-time sequencing

Annealing of the sequencing primer and binding polymerase P6 to the SMRTbell templates were performed in line with the recommendations of the PacBio calculator. The polymerase-template complexes were bound to MagBeads, loaded onto SMRTcells and sequenced on the PacBio RS II sequencer. In brief, the sequencing primer was diluted to 150 nM in PacBio Elution Buffer (EB). The annealing was carried out with 1 μl library DNA (cc: 24ng/μl), the diluted primer and 10x primer buffer. DNA polymerase was diluted to a concentration of 50 nM by Binding Buffer v3 (BB). Diluted polymerase was bound to the annealed template with the following components: dNTP, DTT and BB. The final concentration of the complex was 0.5 nM. Following an incubation at 30 °C for 4 h, 0.5 μl from the sample complex and 18.5 μl MagBead Binding Buffer were mixed (the final concentration was 0.0125 nM). The sample complex was bound to the washed, prepared MagBeads for loading to the RSII sequencer: sample complex was added to the beads, and then it was incubated in a rotator at 4 °C for 30 min. After incubation, the MagBead-bound complex was washed with Bead Binding Buffer, then with Bead Wash Buffer and resuspended in Bead Binding Buffer. The total amount of the MagBead-bound complex was loaded onto the machine. Seven SMRT cells were used for sequencing the poly(A)^+^ library and one for the random primer-based library.

### Transcript annotation

Consensus reads were generated following the PacBio ReadsOfInsert protocol (SMRT Analysis, version v2.3.0), which were then mapped using GMAP^42^. Transcripts were mapped to a modified version of the HCMV strain Towne genome (FJ616285), this modified genome was created by substituting the 180,887–191,406 region of the original genome with the 1–11,996 region. This modified genome, with the transcriptome profiling results is available at the European Nucleotide Archive under the accession number: LT907985. Low-quality reads (mismatch or indel ratio > 5%) were discarded from the analysis. The orientation of the reads was determined based on the presence of an intact 5′ adapter (2 mismatches were allowed) or a poly(A) tail (at least 15 consecutive [A]s). Reads in which both ends contained poly(A) tails or the 5′ adapter sequence, were discarded. In order to identify potential transcription end sites (TESs), polyadenylated reads were considered if the reference genome contained a stretch shorter than 3 (A)s at the 3′ end of the reads. A genomic position was called a TES if at least two reads had their 3′ ends at the exact same nucleotide. Transcription start sites (TSS) were accepted if the number of reads (at least 2) that had a 5′ end at a given nucleotide was significantly higher than at other nucleotides in the same given region. The significance was based on the Poisson-probability (Poisson[k_0_;λ]) of k_0_ read starting at a given nucleotide in a 101-nt-long region where on average $\lambda =\frac{\sum _{i=-50}^{50}{k}_{i}}{101}$ reads ended. Splice junctions were accepted if at least two independent reads (different library preparation, different TSS or TES) contained the same splice junction and the intron started with GT and ended with AG. Transcripts were annotated if there was at least one read that connected an annotated TSS with an annotated TES. Mapping errors were screened for manually, using the Integrative Genomics Viewer 2.3^43^. The Geneious^44^ programme suite was used for importing annotations and for the visualization of the transcriptional landscape of the virus. Since there is no universally accepted nomenclature for transcript isoforms, we applied the same naming convention for the HCMV isoforms that we had used for other viruses in our former publications^17,18,45^. According to this convention, in the names of polycistronic transcripts all the transcribed genes are listed in a 5′ to 3′ order, 5′ UTR isoforms shorter or longer than the previously annotated transcripts are marked with an ‘S’ or and ‘L’ suffix respectively, while 3′ variants are marked with an ‘AT’ suffix, and in the case of splice variants, the name of the transcript is followed by ‘NSP’ (in case of the non-spliced isoforms) or ‘SP’.

### Analysis of the coding potential of HCMV transcript isoforms

Transcript coordinates from our annotation were transferred to the NC_006273.2 (strain Merlin) genome to allow for comparison with the translationally active ORFs, as determined by Stern-Ginossar *et al.* ^7^. The criterion for transferring an annotation was an 85% identity match.

### PCR analysis

The genomic rearrangement of the virus was confirmed by PCR. DNA was isolated, using DNeasy Blood and Tissue Kit (Qiagen) according to the manufacturer’s instructions, followed by amplification using Veriti Thermal Cycler (Applied Biosystems), with KAPA HiFi Enzyme (Kapa Biosystems). Following a 3 min initialization step at 95 °C, 30 PCR cycles were carried out at 92 °C for 30 s (denaturation), at 60 °C for 30 s (annealing), and at 72 °C for 10 s (extension). The final elongation at 72 °C was set to 10 min. The sequences of the primers are listed in Supplementary Dataset S8.

### Prediction of *cis*-regulatory sequences of the HCMV genes

Canonical poly(A) signals^33^ were searched for 1 to 50 nt upstream of each determined TES, for each TES the most common poly(A) signal sequence was annotated. TATA boxes upstream (1–50 nt) of TSSs, were determined using the JASPAR POLII database^46^ and FIMO (Find Individual Motif Occurrences) software^47^ with a threshold of p < 0.0001.

### Conservation of the novel transcripts

To compare the conservation of the novel transcripts, the genomic sequences of novel transcripts were aligned to all (285) human cytomegalovirus complete genome sequences using BLAST^48^. If a novel transcript had multiple isoforms, the longest isoform was selected for the BLAST search. An identity matrix was generated of the BLAST results using MAFFT Alignment^49^ with default settings (gap open penalty = 1.53, offset value 0.123). The column of this matrix containing comparisons to the FJ616285 genome was used to perform the analysis of variance or to create the boxplots.

## Electronic supplementary material

Supplementary Dataset 1

Supplementary Dataset 2

Supplementary Dataset 3

Supplementary Dataset 4

Supplementary Dataset 5

Supplementary Dataset 6

Supplementary Dataset 7

Supplementary Dataset 8

Supplementary Info
