# Supplementary material for: Long-Read Sequencing of Human Cytomegalovirus Transcriptome Reveals RNA Isoforms Carrying Distinct Coding Potentials
Source: Sci Rep. 2017 Nov 22;7:15989. doi: 10.1038/s41598-017-16262-z (PMC5700075; doi:10.1038/s41598-017-16262-z)
Supplement: Supplementary file 9 — Supplementary Info [file 41598_2017_16262_MOESM9_ESM.pdf]

Long-Read Sequencing of Human Cytomegalovirus Transcriptome Reveals RNA Isoforms Carrying Distinct Coding Potentials

Supplementary Material

Zsolt Balázs[1†], Dóra Tombácz[1,2†], Attila Szűcs[1], Zsolt Csabai[1], Klára Megyeri[3], Alexey N. Petrov[4,5], Michael Snyder[2], Zsolt Boldogkői*[1]

[1]Department of Medical Biology, Faculty of Medicine, University of Szeged, Szeged, 6720, Hungary

[2]Department of Genetics, School of Medicine, Stanford University, Stanford, California, 94305, USA

[3]Department of Medical Microbiology and Immunobiology, Faculty of Medicine, University of Szeged, Szeged, 6720, Hungary

[4]Department of Structural Biology, School of Medicine, Stanford University, Stanford, California, 94305, USA

[5]Department of Biological Sciences, College of Sciences and Mathematics, Auburn University, Auburn, Alabama, 36849, USA

[†]these two authors contributed equally to this work

* To whom correspondence should be addressed

Tel: +36-62-545-595

Fax: +36-62-545-131

E-mail: boldogkoi.zsolt@med.u-szeged.hu (ZBo)

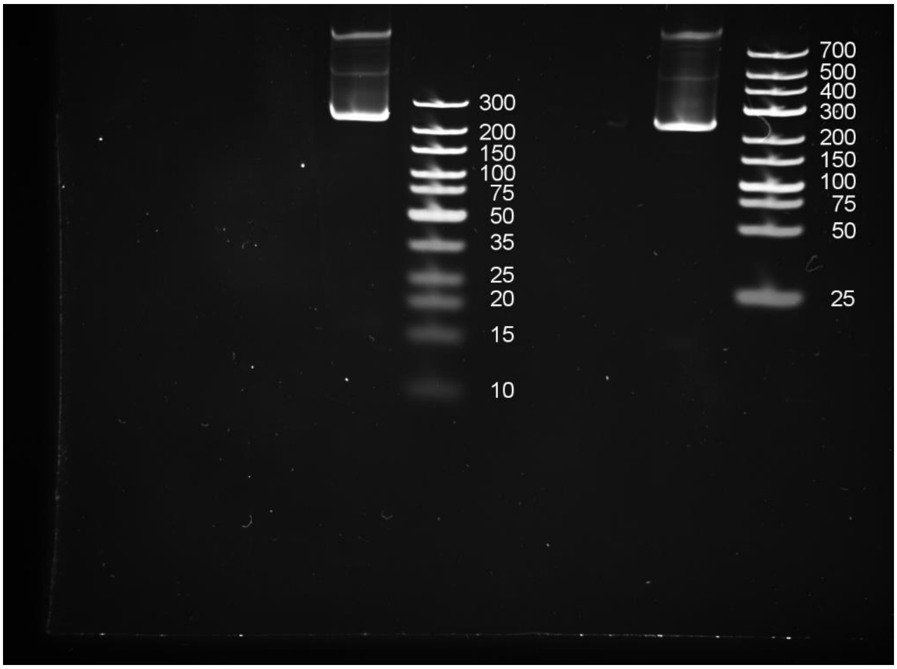

Supplementary Figure S1. The original gel photo used to create Fig. 1 Panel B. Molecular-weight size markers for GeneRuler Ultra Low Range DNA Ladder (left) and for GeneRuler Low Range DNA Ladder (right) are annotated. The image was cropped, and rotated to the left to produce the picture in Fig. 1.

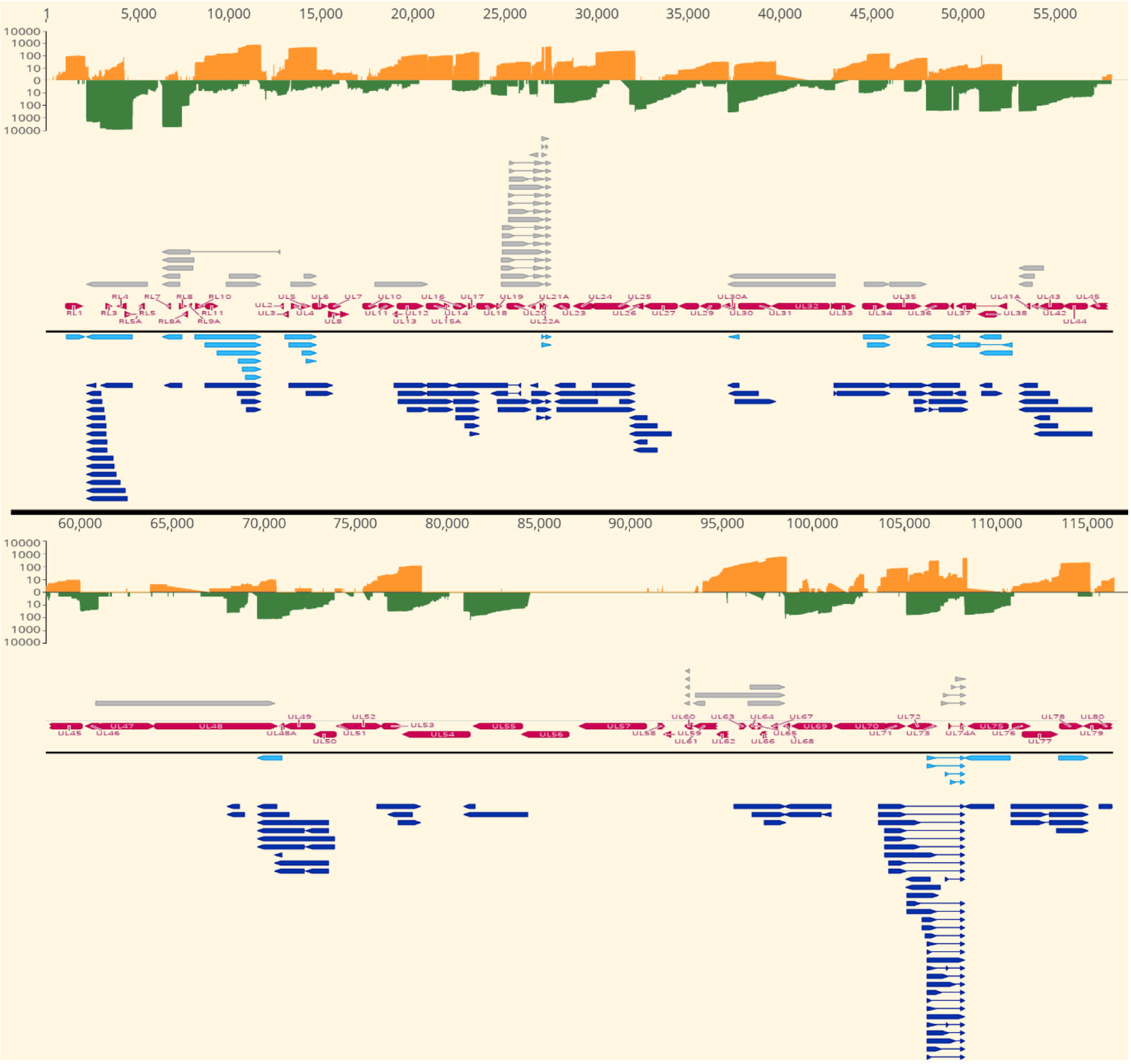

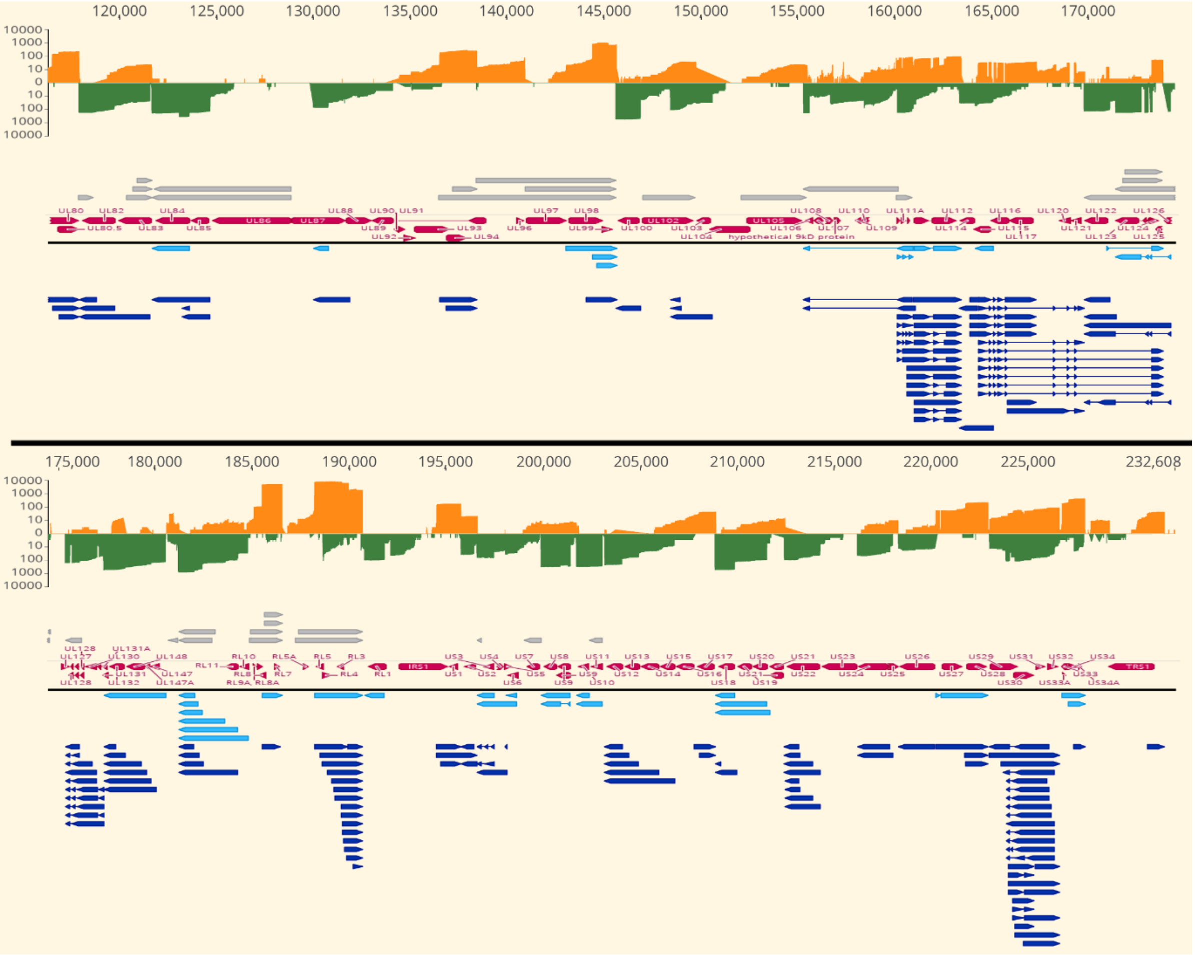

Supplementary Figure S2. Transcriptional landscape of the HCMV. (continued from the previous page) The figure presents the coverage and the annotated transcripts on the genome in four blocks. The coverage histogram (above) is drawn on a logarithmic scale, where the orange bars represent coverage on the plus strand and dark green bars represent coverage on the minus strand. The coverage values of oriented reads from the random and poly(A)$^{+}$ libraries are summed. The annotated transcripts are located below the coverage chart. The previously described transcripts that were not detected by our RNA-sequencing are grey, while the previously described transcripts that our experiments have confirmed have been depicted in light blue, and novel transcript isoforms have been labelled with dark blue. Between the transcripts, canonical ORFs are indicated with purple.

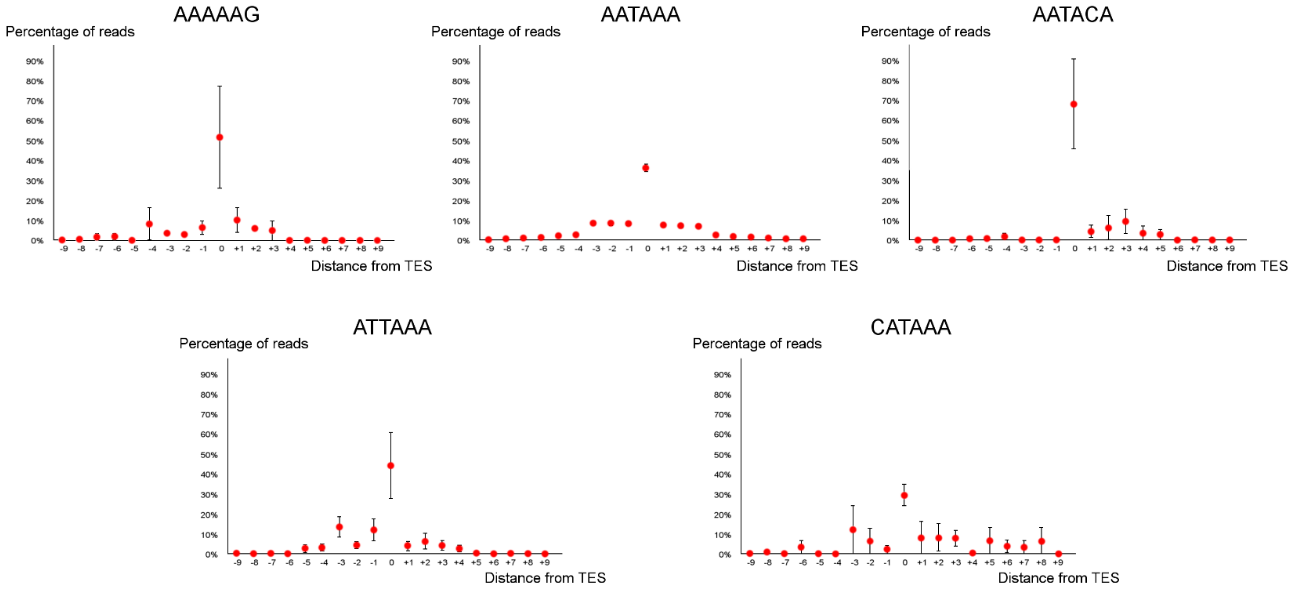

Supplementary Figure S3. Different polyadenylation signals lead to different read ending distribution around TESs. The scatter plot shows the frequency of reads ending in the vicinity of a TES depending on the sequence of the polyadenylation signal. Error bars represent standard errors.

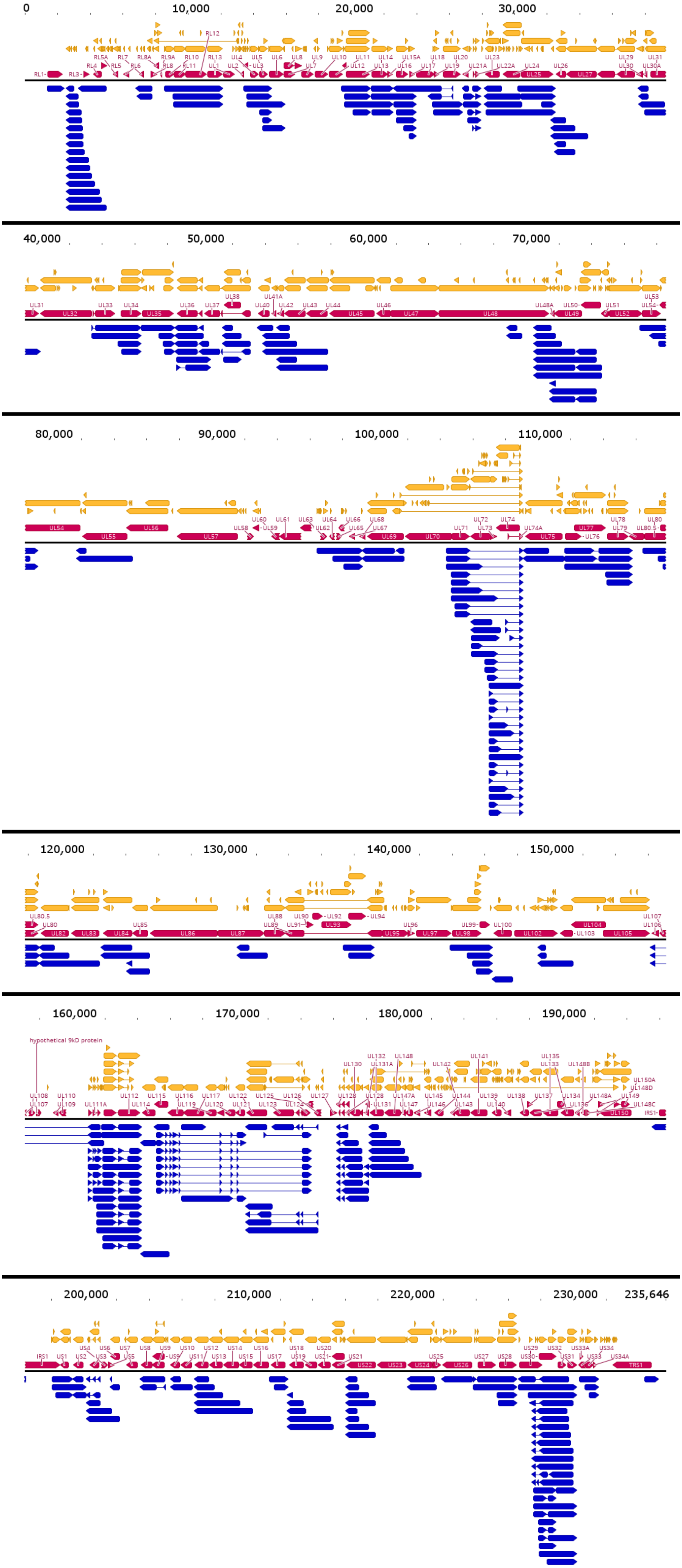

Supplementary Figure S4. The annotated transcripts and translationally active ORFs. The transcripts identified by our sequencing experiments (blue) and the ORFs with a significant ribosome footprint as described by Stern-Ginossar et al.[7] (orange) are depicted on the genome map of HCMV strain Merlin (NC_006273). In the middle, canonical ORFs are indicated with purple.

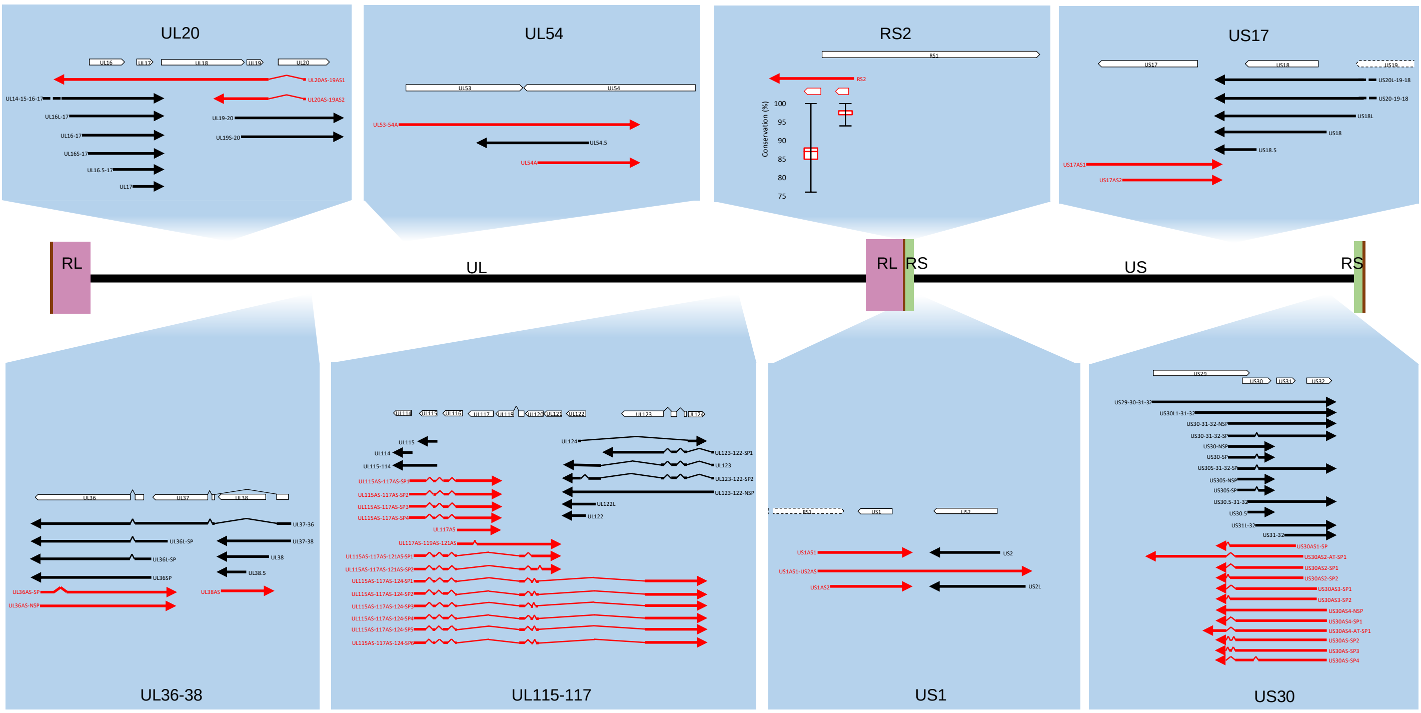

Supplementary Figure S5. Schematic representation of the genomic regions containing the novel transcripts. The HCMV genome is situated in the middle with brown, pink and green rectangles marking the repeat sequences a, b, and c, respectively. The genomic regions containing the novel transcripts are magnified. Canonical ORFs (thick, black arrows) are displayed above the identified transcripts (thin arrows). Novel transcripts are depicted as red. The boxplot shows the nucleotide conservation of the two (not canonical) ORFs found in the transcript RS2 (thick, red arrows). The whiskers represent the range of the data.

Supplementary Dataset S1. Genomic regions that produce artificially 3'-truncated transcripts. Polyadenylated reads terminated at the listed genomic positions, which contain stretches of 3 or more (A)s and therefore they are likely to be false TESs. Coordinates are referenced according to the LT907985 HCMV Towne genome.

Supplementary Dataset S2. Transcriptional end sites. Annotated TESs and the numbers of reads confirming these are listed along with the corresponding polyadenylation signals. Polyadenylation signals were predicted in the genomic sequences 1-50 nt upstream of the TESs. TESs that belonged to an annotated transcript carries their respective names, whereas TESs without corresponding transcripts have been numbered. Coordinates are referenced according to the LT907985 HCMV Towne genome.

Supplementary Dataset S3. Transcriptional start sites. Annotated TSSs and the numbers of reads confirming them are listed along with their distance from the nearest TATA boxes (if applicable). TATA boxes were predicted in the genomic sequences 1-50 nt upstream of the TSSs. TSSs that had belonged to an annotated transcript, carry its name, whereas TSSs without a corresponding transcript numbered. Coordinates are referenced according to the LT907985 HCMV Towne genome.

Supplementary Dataset S4. Deletions breaking the GT/AG rule. The genomic positions of intron-like deletions that did not adhere to the GT/AG rule are listed along with the nucleotide sequences of the donor and acceptor sites. All of these deletions contained 3-6-nt-long repeat sequences (occurring upstream of the donor and downstream of the acceptor sites or downstream of the donor and upstream of the acceptor sites). Coordinates are referenced according to the LT907985 HCMV Towne genome.

Supplementary Dataset S5. Splice junctions. Annotated splice junctions are listed, as well as repeat sequences that may serve as catalysts for template switching. The numbers of reads confirming a given splice site are given for the random and the poly(A) selected libraries as well. Reference numbers for known splice junctions indicate publications where they have been described. Coordinates are referenced according to the LT907985 HCMV Towne genome.

Supplementary Dataset S6. HCMV transcripts and transcript isoforms. Transcript locations are given in GenBank format to include not only TSSs and TESs but also strand and intronic data. The number of reads that contained all features of a transcript isoform (TSS, TES and all splice junctions) are also included along with a relative abundance expressed in percentage compared to the total number of annotated reads. In the case of splice isoforms where this number is 0, the splicing patterns were determined by reads that never reached both the TSS and the TES at the same time. Reference numbers for known transcripts indicate publications where they have been described. Coordinates are referenced according to the LT907985 HCMV Towne genome.

Supplementary Dataset S7. Primers used in the study. The table lists the primer sequences used for the confirmation of the genomic rearrangement and for the RT-qPCR analyses.

References used in the Supplementary Material

1. Gatherer, D. *et al.* High-resolution human cytomegalovirus transcriptome. *Proc. Natl. Acad. Sci. U. S. A.* **108,** 19755–60 (2011).
2. Stern-Ginossar, N. *et al.* Decoding human cytomegalovirus. *Science* **338,** 1088–93 (2012).
3. Ma, Y. *et al.* Human CMV transcripts: an overview. *Future Microbiol.* **7,** 577–593 (2012).
4. Gao, S. *et al.* Newly identified transcripts of UL4 and UL5 genes of human cytomegalovirus. doi:10.18388/abp.2014_844
5. He, R. *et al.* Characterization of human cytomegalovirus UL146 transcripts. *Virus Res.* **163,** 223–228 (2012).
6. Scott, G. M., Barrell, B. G., Oram, J. & Rawlinson, W. D. Characterisation of Transcripts from the Human Cytomegalovirus Genes TRL7, UL20a, UL36, UL65, UL94, US3 and US34. *Virus Genes* **24,** 39–48 (2002).
7. Gao, S. *et al.* Validation of three splice donor and three splice acceptor sites for regulating four novel low-abundance spliced transcripts of human cytomegalovirus UL21.5 gene locus. *Int. J. Mol. Med.* **35,** 253–262 (2015).
8. Nelson, P. N. *et al.* A polymerase chain reaction to detect a spliced late transcript of human cytomegalovirus in the blood of bone marrow transplant recipients. *J. Virol. Methods* **56,** 139–148 (1996).
9. Weston, K. An enhancer element in the short unique region of human cytomegalovirus regulates the production of a group of abundant immediate early transcripts. *Virology* **162,** 406–16 (1988).
10. Zheng, B. *et al.* Characterization of a novel group of antisense transcripts in human cytomegalovirus UL83 gene region. *J. Med. Virol.* **86,** 2033–2041 (2014).
11. Jenkins, C., Abendroth, A. & Slobedman, B. A novel viral transcript with homology to human interleukin-10 is expressed during latent human cytomegalovirus infection. *J. Virol.* **78,** 1440–7 (2004).
12. Yang, C.-Q. *et al.* Natural antisense transcripts of UL123 packaged in human cytomegalovirus virions. *Arch. Virol.* **159,** 147–151 (2014).
